# Association of Ex-Confederate Medical Officers

**Published:** 1874-05

**Authors:** 


*Association of ex-Confederate Medical Officers.*

This Association was organized in Atlanta, Ga., the 20th of May. The attendance was not as large as was expected or desired, but sufficient progress was made to give assurance of success for the future. Late Surgeon General S. P. Moore, of Richmond, Va., was elected President. One Vice-President at large, and one from each of the late Confederate States. Among the latter occur the names of Dr. S. S. Satchwell, of North Carolina, and Dr. V. D. S. Hill, of Mississippi, both of these gentlemen being members of the editorial staff of the RECORD. The crowded condition of our pages must be our excuse for this meager notice of a very important movement for the collection of medical and scientific facts, which otherwise might be lost to the profession. The Association meets at Richmond, Va., on the first Wednesday of July, 1875. G.

